# A compact topic: How ethylene controls crown root development in compacted soil

**DOI:** 10.1093/plcell/koae091

**Published:** 2024-03-25

**Authors:** Gwendolyn K Kirschner

**Affiliations:** Assistant Features Editor, The Plant Cell, American Society of Plant Biologists; The James Hutton Institute, Invergowrie, Dundee DD2 5DA, UK

Compacted soil is an often-overlooked problem in agriculture, as it does not show at the soil surface but rather occurs under the tilled soil layers. It is considered one of the biggest environmental problems arising from agriculture, caused by the deployment of heavy machinery and by tillage practices (Hamza and Anderson 2005). Soil compaction limits the availability and transport of water and nutrients and makes the penetration of roots into deeper soil layers more difficult (Hamza and Anderson 2005). Recently, it was shown that roots can sense the level of soil compaction through the volatile hormone ethylene: compacted soil reduces gas diffusion through a reduction of air-filled pores. This hinders the diffusion of ethylene, which then accumulates in the root tissue, resulting in an inhibition of root growth (Pandey et al. 2021). At the same time, ethylene is also known as a developmental signal promoting the outgrowth of crown roots in rice (Lin and Sauter 2018).

Yuxiang Li, Juan Wang, and colleagues **(Li et al. 2024)** now connect the 2 roles of ethylene: as a signal responding to soil compaction and to trigger crown root development. In this work, they mimicked different levels of compaction by increasing agar concentrations and compared the growth of wild-type plants and ethylene signaling mutants. They found that compaction triggered crown root initiation via an ethylene-dependent pathway (Fig. 1A), and manipulating ethylene-related regulators influenced both root development and grain yield, implying that these regulators balance both root and grain development.

**Figure 1.**
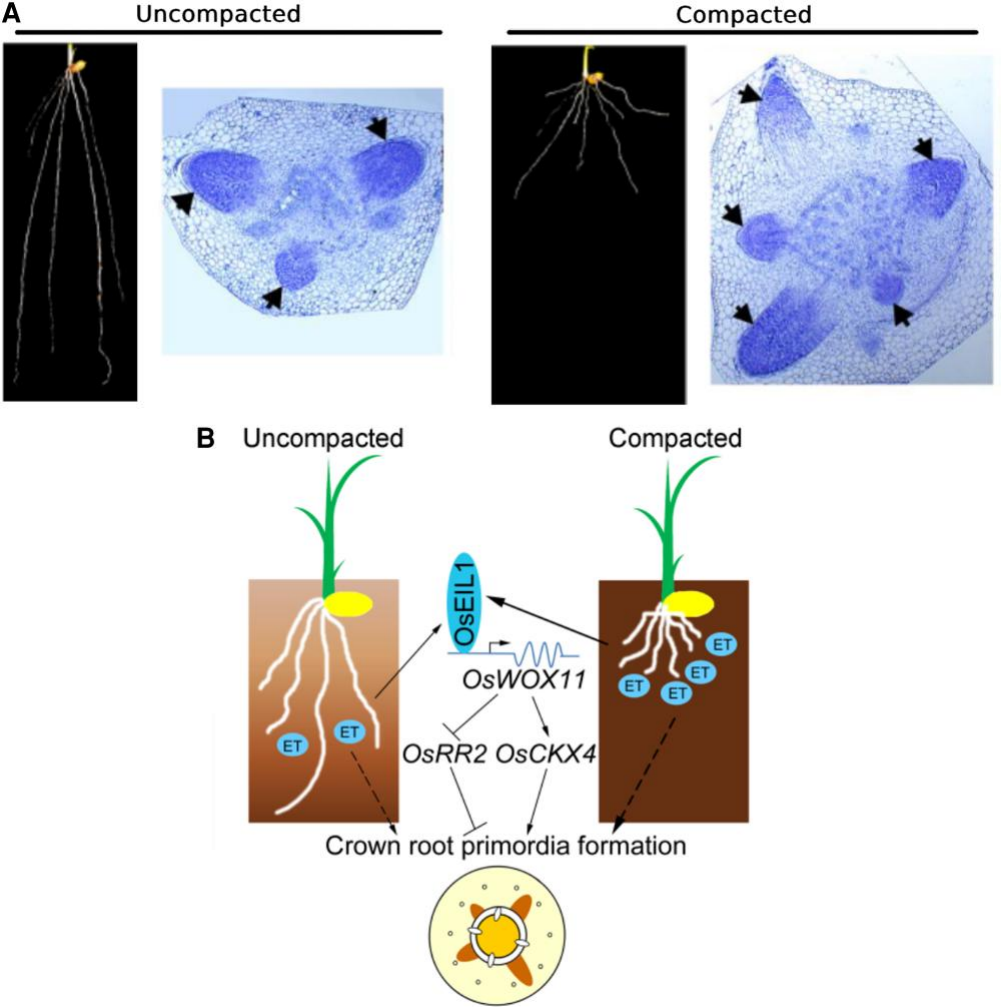
Regulation of rice crown root development under compacted soil conditions. **A)** Compared with uncompacted soil conditions (left), rice crown root number increases under compacted soil conditions (right). **B)** Compacted soil leads to an accumulation of ethylene (ET) and increased OsELI1 transcript levels in the roots, which in turn increases OsWOX11 transcription. OsWOX11 regulates OsRR2 and OsCKX4, which then promotes crown root primordium initiation and development. Adapted from Li et al. (2024), Figures 1 and 7.

By analyzing the transcriptome of an ethylene signaling mutant, the authors then identified *WUSCHEL-related Homeobox 11* (*OsWOX11*) as an ethylene-regulated gene. *OsWOX11* is a known activator of crown root emergence and growth, and accordingly, the *oswox11* mutant had fewer crown roots (Zhao et al. 2009). However, it also showed a slight reduction in grain length and width compared to the wild type, while the opposite effects occurred in *OsWOX11* overexpressing lines. Ethylene treatment and growth of the mutant and overexpressing lines in medium mimicking compacted soil showed that *OsWOX11* was required for ethylene-stimulated crown root development, as well as for compaction-stimulated crown root development.

It was previously shown that OsWOX11 directly activates the expression of the type-A cytokinin-responsive regulator gene *OsRR2* and *CYTOKININ OXIDASE 4* (*OsCKX4*) during crown root development (Zhao et al. 2009). Li et al. (2024) showed that this regulation occurred downstream of ethylene signaling. Using chromatin immunoprecipitation in combination with electrophoretic mobility shift and luciferase assays, the authors showed that the transcriptional master regulator of ethylene responses, ETHYLENE INSENSITIVE 3-LIKE 1 (OsEIL1), directly binds to the *OsWOX11* promoter in vitro and in vivo and activates *OsWOX11* expression. By analyzing crown root numbers and transcript levels in *oswox11* and ethylene signaling mutants, or overexpression combinations under normal or compacted soil conditions, the authors confirmed that the ethylene-OsEIL1-OsWOX11 module facilitates crown root development in compacted soil (Fig. 1B).

The findings of Li, Wang, and colleagues show that ethylene promotes crown root development in response to compacted soil. In parallel, ethylene inhibits the elongation of primary roots in response to soil compaction (Pandey et al. 2021), suggesting that ethylene steers root development toward more efficient exploitation of soil resources: it inhibits the elongation of the embryonic roots that have a steeper root growth angle and thereby facilitates avoidance of the deeper, more compact soil layers, while increasing the outgrowth and development of the more horizontally growing crown roots so that the plant can forage the less compacted upper soil layer for nutrients.

## Dive Curated Terms

The following phenotypic, genotypic, and functional terms are of significance to the work described in this paper:

CYTOKININ CHEBI: CHEBI:23530

ethylene treatment Gramene: EO:0007068
ethylene treatment Araport: EO:0007068
